# First dorsal metacarpal artery flap with dorsal digital nerve with or without dorsal branch of the proper digital nerve produces comparable short-term sensory outcomes

**DOI:** 10.1186/s13018-021-02838-z

**Published:** 2021-11-18

**Authors:** Shi-Ming Feng, Jia-Ju Zhao, Filippo Migliorini, Nicola Maffulli, Wei Xu

**Affiliations:** 1 Department of Orthopaedics, The Second Affiliated Hospital of Soochow University, No. 1055, the Sanxiang Road, Suzhou, 215004 Jiangsu People’s Republic of China; 2 Department of Orthopaedics, Xuzhou Central Hospital, Xuzhou Clinical College of Xuzhou Medical University, Xuzhou, 221009 Jiangsu People’s Republic of China; 3 Department of Orthopaedics, Trauma, and Reconstructive Surgery, RWTH University Hospital Aachen, Pauwelsstraße 30, 52074 Aachen, Germany; 4 Department of Musculoskeletal Disorders, Faculty of Medicine and Surgery, University of Salerno, Salerno, Italy; 5 Guy Hilton Research Centre, School of Pharmacy and Bioengineering, Keele University, Stoke-on-Trent, Staffordshire ST4 7QB England; 6 Centre for Sports and Exercise Medicine, Barts and The London School of Medicine and Dentistry, Mile End Hospital, 275 Bancroft Road, London, E1 4DG England

**Keywords:** First dorsal metacarpal artery flap, Dorsal digital nerve, Dorsal branches of the proper digital nerve

## Abstract

**Background:**

The first dorsal metacarpal artery flap, including dorsal digital nerves with or without dorsal branches of the proper digital nerves, can be used to reconstruct thumb pulp defects with good results. However, it is still unclear whether there are differences in the sensory outcomes between preserving or not preserving the dorsal branches of the proper digital nerves.

**Methods:**

This retrospective cohort study included 137 thumb pulp defect patients who underwent first dorsal metacarpal artery flap reconstruction procedure from October 2015 to June 2019. Patients were divided into two groups according to whether the dorsal branches of the proper digital nerves were preserved. In the non-preservation group (*n* = 80), the dorsal digital nerves were included in the flap for sensory reconstruction. In the preservation group (*n* = 57), the dorsal digital nerves and the dorsal branches of the proper digital nerves of the index finger were included in the flap. The stump of the proper digital nerves in the defect was coaptated to the donor nerves of the flap using the end-to-end fashion. At the last follow-up, static two-point discrimination, Semmes–Weinstein monofilament scores, pain, cold intolerance of the reconstructed finger, and patient satisfaction in both groups were compared.

**Results:**

All patients were followed up for at least 17 months. No significant differences were found regarding pain of thumb pulp, static two-point discrimination, Semmes–Weinstein monofilament score, cold intolerance in the injured finger, and patient satisfaction. The non-preservation group presented slightly shorter operative times (*p* < 0.05).

**Conclusion:**

There are no differences at 2 years in postoperative clinical outcomes when dorsal digital nerves are used to reconstruct flap sensation regardless of preservation of the dorsal branches of the proper digital nerves in the first dorsal metacarpal artery flap.

*Level of evidence*: Level III, retrospective comparative study.

## Background

**Fig. 1:**
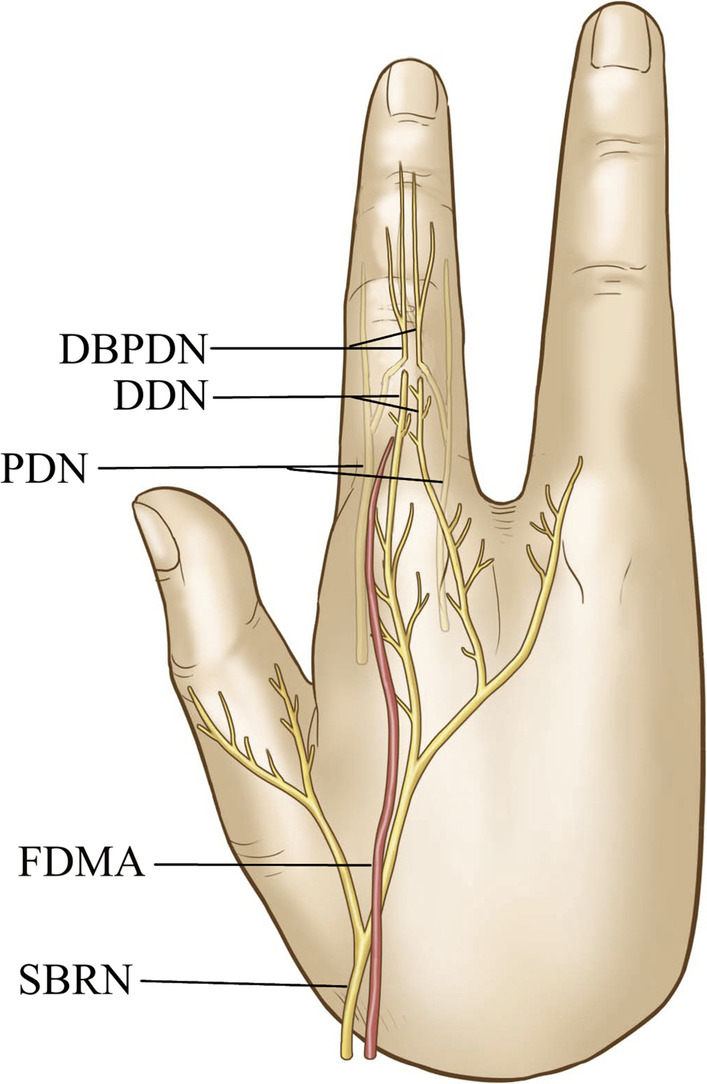
Diagram showing that the dorsal digital nerves (DDN) and the dorsal branches of the proper digital nerves (DBPDNs) dominate the dorsal skin sensation of the index finger proximal phalanx. The DDN arises from the superficial branch of the radial nerve (SBRN), and the DBPDNs originate from the proper digital nerves (PDNs). The first dorsal metacarpal artery (FDMA) dominates the dorsal skin nutrition of the index finger proximal phalanx

The first dorsal metacarpal artery flap is currently the first choice for the treatment of thumb pulp defect [1–3]. Restoration of the sensory function of the thumb pulp is a major challenge for hand surgeons. The superficial branches of the radial nerve, which divides into the dorsal digital nerves, or the dorsal branches of the proper digital nerves of the index finger are the most commonly used nerves with the first dorsal metacarpal artery flap to restore the sensation of the thumb pulp (Fig. 1) [4–6].

In thumb pulp reconstruction with the first dorsal metacarpal artery flap, it remains unclear whether preserving the dorsal branches of the proper digital nerve at surgery is advantageous. To our knowledge, no report demonstrates whether dorsal branches of the proper digital nerve preservation produced superior outcomes to non-preservation procedures in first dorsal metacarpal artery flap with dorsal digital nerves.

We compared the clinical outcome between dorsal branches of the proper digital nerve preservation and non-preservation in terms of thumb function, sensory outcomes, and patient satisfaction.

## Methods

### Patient selection

This investigation was a retrospective cohort study evaluating the clinical results of the patients undergoing modified first dorsal metacarpal artery flap for thumb pulp reconstruction with or without dorsal branches of the proper digital nerves of the index finger. Our institutional ethics review boards approved the study. All patients provided a signed informed consent as well as consent under the Health Insurance Portability and Accountability Act to participate in this study. Inclusion criteria were: (1) thumb pulp defect patients underwent modified first dorsal metacarpal artery flap; (2) the dorsal digital nerves (superficial branches of the radial and ulnar nerves of the index finger) were included in the flap with or without the dorsal branches of the proper digital nerves of the index finger; (3) complete surgical and follow-up data; and (4) follow-up time was not less than 17 months. Exclusion criteria were: (1) combined multi-finger injury; (2) combined thumb fracture or tendon rupture; and (3) previous finger injury or surgery on the affected hand, or secondary finger injury during the follow-up period.

### Patients’ information

From October 2015 to June 2019, 252 consecutive patients with a thumb pulp defect underwent modified first dorsal metacarpal artery flap by a senior surgeon with extensive experience in hand surgery. A total of 115 patients did not meet the inclusion criteria and were excluded. The common reasons for exclusion were thumb bone fracture (*n* = 54), thumb tendon injury (*n* = 30), multi-finger injury (*n* = 22), and secondary finger injury during the follow-up period (*n* = 9). A total of 137 thumb pulp defect patients undergoing modified first dorsal metacarpal artery flap were enrolled into the current study according to the above inclusion and exclusion criteria.

The patients selected the surgical procedure according to their own wishes after communicating with the doctor. Among the recruited 137 patients, 80 underwent modified first dorsal metacarpal artery flap carrying dorsal digital nerves of the index finger and the other 57 were treated with modified first dorsal metacarpal artery flap carrying dorsal digital nerves and dorsal branches of the proper digital nerves of the index finger. The two groups were comparable in terms of sex, age, defect area, flap size, and follow-up time (Table 1).

**Table 1:** Patient demographics (VAS, visual analogue scale)

Variable	Preservation group (*n* = 57)	Non-preservation group (*n* = 80)	p*
Age, yr	34.81 ± 9.06	35.08 ± 11.01	0.880^†^
Sex			0.790^$^
Male	39 (68.4%)	53 (66.3%)	
Female	18 (31.6%)	27 (33.7%)	
VAS (pain of thumb pulp)	6.16 ± 0.75	6.14 ± 0.76	0.876^†^
Pulp defect, cm	8.15 ± 2.25	8.71 ± 2.48	0.180^†^
Flap size, cm	9.27 ± 2.38	9.84 ± 2.60	0.189^†^
Follow-up time, mo	25.35 ± 4.92	24.53 ± 5.63	0.375^†^

*A value *p* < 0.05 was set as statistically significant

^†^*t* test

^$^Pearson χ^2^ test

### Surgical techniques

**Fig. 2:**
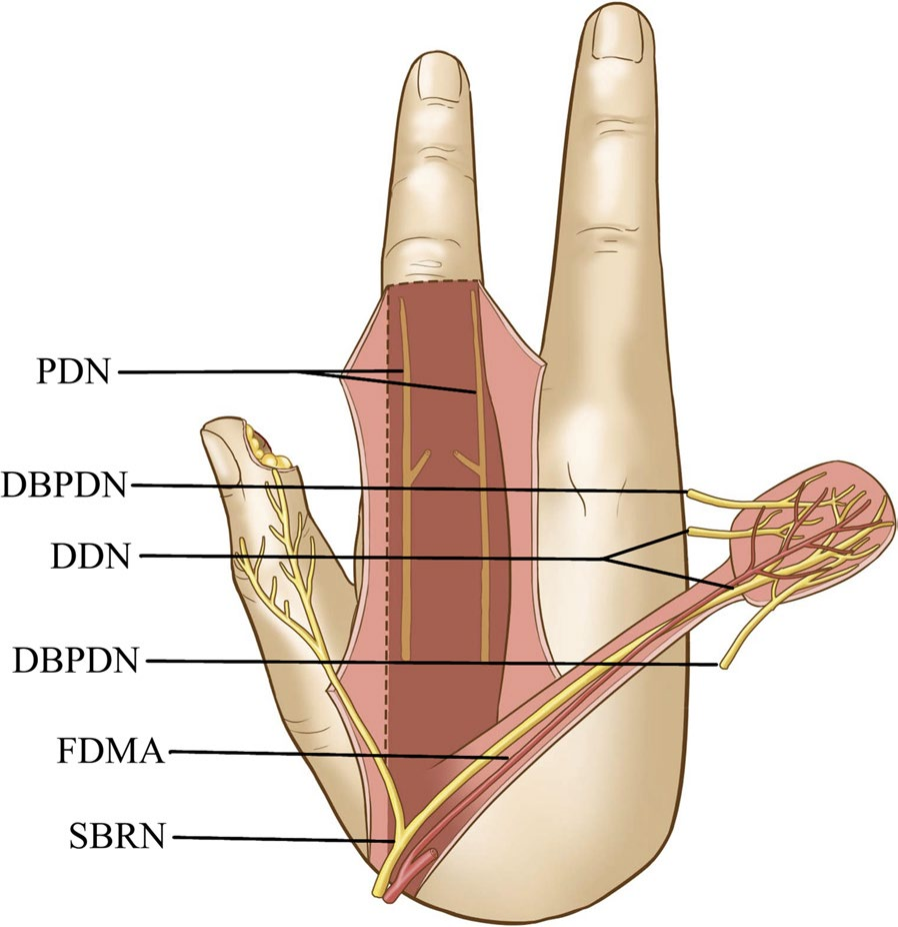
Diagram showing that the modified first dorsal metacarpal artery flap in which the dorsal branches of the proper digital nerves (DBPDNs) were preserved with, including two dorsal branches of the DBPDNs and the dorsal digital nerves (DDNs), is raised based on the first dorsal metacarpal artery (FDMA). The DDN arises from the superficial branch of the radial nerve (SBRN)

Preservation group: The flap was designed according to the shape and size of the thumb pulp defect. On the dorsum of the proximal phalanx of the index finger, the flap was raised along its margins. The integrity of the superficial branch of the radial nerve was maintained. The superficial branch of the radial nerve in the pedicle and an 8-mm-wide strip of subcutaneous tissue around the pedicle were preserved. The ulnar dorsal branch of the index finger of the superficial branch of the radial nerve (dorsal digital nerve) was raised with the flap. The dorsal branches of the proper digital nerves of the index finger were incised, with its integrity maintained in the flap. Through a subcutaneous tunnel, the raised flap was transferred to the wound, with the dorsal digital nerve and dorsal branches of the proper digital nerves of the index finger coaptated to the stump of the proper digital nerves in the wound in end-to-end fashion (Fig. 2).

**Fig. 3:**
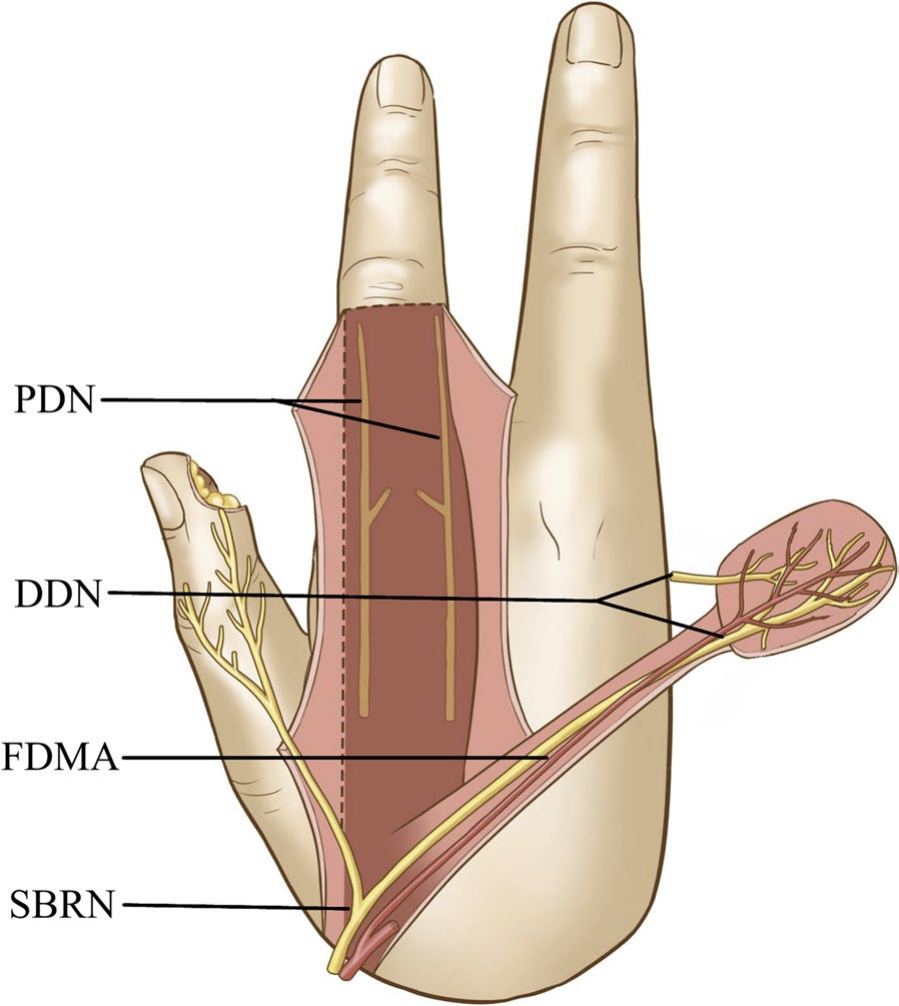
Diagram showing that the modified first dorsal metacarpal artery flap in which the dorsal branches of the proper digital nerves (DBPDNs) were not preserved, including the dorsal digital nerves (DDNs) arising from the superficial branch of the radial nerve (SBRN), is raised based on the first dorsal metacarpal artery (FDMA)

Non-preservation group: The flap was designed and raised as was the case in the preservation group. The superficial branch of the radial nerve was preserved in the flap and pedicle. The ulnar dorsal branch of the index finger of the superficial branch of the radial nerve (dorsal digital nerve) was raised with the flap and coaptated to the stump of the proper digital nerves in the wound in end-to-end fashion. The dorsal branches of the proper digital nerves of the index finger were not carried with the flap. The wound was covered with the flap without tension (Fig. 3).

### Postoperative management

After surgery, the injured hand was placed above heart level to reduce possible flap venous congestion. Flap circulation was monitored closely for at least 24 h. The patients routinely received antibiotics and oral nonsteroidal anti-inflammatory drugs for 3 days after the procedure. On the second day after the surgery, patients were instructed to carry out early isometric exercise of forearm muscles. A splint was applied on the thumb to allow the flap to settle. Two weeks after the surgery, the splint was removed, and gentle mobilization started.

### Evaluation of outcomes

The patients were informed that they would be examined in the future when they were discharged. The appearance of the reconstructed finger and the donor site were assessed using the Michigan Hand Outcomes Questionnaire (MHQ) [7]. The sensory outcomes of the flap were evaluated using the static two-point discrimination (2-PD) [8] and Semmes–Weinstein monofilament (SWM) scores [9]. The visual analogue scale (VAS) was used to evaluate the pain of the injured and donor fingers. The results of the VAS score were divided into three degrees (mild, moderate, and severe) corresponding to three ranges (0–3 cm, 4–6 cm, and 7–10 cm). Cold intolerance of the flaps was measured using the self-administered Cold Intolerance Severity Score questionnaire (the maximum score is 100; grouped into 0–25, 26–50, 51–75, 76–100, corresponding to mild, moderate, severe, and extreme severity, respectively) [10]. All measurements were undertaken by the same experienced hand surgeon who was blinded to the procedure performed.

### Statistical analysis

The SPSS 19.0 statistical software (SPSS, Inc., Chicago, IL, USA) was used for analysis. The measurement data (e.g., VAS, 2-PD, SWM, and MHQ), in each group before and after surgery, as well as between two groups after surgery, were compared and analyzed using the t test (symmetric distribution) or the Mann–Whitney test (asymmetric distribution). Pearson Chi-square test was used to evaluate associations among nominal categorical variables. Significance level was set at 5%, and *p* < 0.05 was considered statistically significant. The α value was set as 0.05 given the univariate comparisons before and after surgery. A post hoc power analysis was performed.

## Results

Full flap survival was achieved in both groups. All the wounds healed uneventfully, with no wound complication, no nervous complication, or venous congestion in the flap. The patients in both groups were discharged 3–7 days after the index procedure. The operative time in the preservation group was 97.46 min (range 81–119 min) and 74.34 min (range 55–100 min) in the non-preservation group (*p* < 0.001). The hospital stay duration was comparable between the two groups (Table 2).

**Table 2:** Comparison of the two groups (2-PD, two-point discrimination; SWM, Semmes–Weinstein monofilament; VAS, visual analogue scale)

Variable	Preservation group (*n* = 57)	Non-preservation group (*n* = 80)	p*	Power‡
Operative time, min	97.46 ± 9.50	74.34 ± 10.67	< 0.001^†^	
Hospital stay duration, days	4.53 ± 1.18	4.59 ± 1.20	0.767^†^	0.060
2-PD of pulp, mm	6.67 ± 1.26	6.89 ± 1.22	0.306^†^	0.173
SWM of pulp, g	3.91 ± 0.38	3.85 ± 0.44	0.433^†^	0.135
Cold intolerance			0.637^$^	
No	37 (64.9%)	55 (68.8%)		
Mild	20 (35.1%)	25 (31.2%)		
VAS (pain of thumb pulp)	0.00 (0.00–1.00)	0.00 (0.00–1.00)	0.222^&^	
Patient satisfaction	4.75 ± 0.43	4.81 ± 0.39	0.416^†^	0.132
Donor site pain			0.892^$^	
No	40 (70.2%)	57 (71.2%)		
Mild	17 (29.8%)	23 (28.8%)		

*A value *p* < 0.05 was set as statistically significant

^†^*t* test

^$^Pearson χ^2^ test

^‡^Power is computed to reject the null hypothesis of equal means

^&^Mann–Whitney test

At the last follow-up, the average VAS scores in preservation and non-preservation groups were 0.00 points (range 0–1 points) and 0.00 points (range 0–1 points), respectively. The results in both groups were markedly lower than those before surgery, and the intergroup difference was not statistically significant. At the last follow-up, the average static 2-PD of the flap was 6.67 mm (range 4.7–10.5 mm) in the preservation group and 6.89 mm (range 5–10 mm) in the non-preservation group (*p* = 0.306). The average SWM scores in the preservation group and the non-preservation group showed no significant difference at 3.91 (range 3.22–4.56) and 3.85 (range 3.22–4.56), respectively. According to the MHQ, patient satisfaction was 4.75 (range 4–5) in the preservation group and 4.81 (range 4–5) in the non-preservation group, respectively (*p* = 0.416). In the preservation group, 40 patients reported no pain at the donor site and 17 patients mild pain. In the non-preservation group, 57 patients had no donor site pain and 23 patients mild pain. No statistical difference between the two groups was observed. Group sample sizes of 57 and 80 achieve less than 20.00% power (hospital stay duration, 2-PD, SWM, and MHQ, respectively) to reject the null hypothesis of equal means, with a significance level (alpha) of 0.050 using a two-sided two-sample unequal-variance *t*-test.

## Discussion

Thumb pulp plays a fundamental role in grip by virtue of its specialized covering. Various surgical techniques are available for thumb pulp reconstruction [11–15]. Length preservation and sensory recovery are the main factors taken into consideration when selecting reconstruction procedures [16].

Local pedicled flaps are the most commonly used. V–Y advancement flaps provide good tissue coverage and sensory restoration and are used for defects less than 1.5 cm long [17]. For longer defects, neurovascular pedicle flaps are suitable alternatives [18]. However, the original injury might involve the dorsal arteries and cause dorsal skin and nail bed necrosis. An innervated cross-finger flap is an alternative technique for large and complex thumb pulp defect reconstruction, though it requires 2–3 weeks of immobilization and a further surgery [19]. The first dorsal metacarpal artery flap is the most commonly used flap to repair the thumb pulp defect. The conventional first dorsal metacarpal artery flap carrying the superficial branch of the radial nerve has proven useful in the restoration of the sensation of the thumb pulp. Ege et al. [20] studied 21 patients who underwent thumb pulp reconstruction using the first dorsal metacarpal artery flap based on radial nerve-sensitive branches on the dorsum of the second metacarpal and proximal phalanx. In their study, 2-PD of the reconstructed thumb pulp was 10.8 mm (range 8–20). Wang et al. [21] reconstructed thumb pulp defects using the first dorsal metacarpal artery flap carrying the dorsal branches of the radial nerve of the index finger in 25 patients who recovered an average static 2-PD of 10 mm (range 8–12 mm). However, the technique resulted in poor 2-PD in the thumb pulp because the first dorsal digital nerve mainly innervates the radial side of the flap. The modified first dorsal metacarpal artery flap with dorsal branches of the proper digital nerve coaptation was reported for sensory reconstruction in 2004 [22]. The two dorsal branches of the proper digital nerves of the index finger were carried and coaptated to the proper digital nerve stumps of the defect. This modified technique greatly improved the sensation in the flap. Chen et al. [4] described the above technique with both dorsal branches of the proper digital nerve in 11 patients, and the mean values of the static 2-PD were 5 mm (range 4–8 mm) and 6 mm (range 4–8 mm) on the radial and ulnar sides of the distal portion of the flap, respectively. We studied 31 patients who underwent the above technique and obtained an average static 2-PD of 7.1 mm (range 5–11 mm) [23]. However, the nerve dissection and coaptation required longer operative time compared to conventional technique.

The dorsal digital nerve is commonly used in island flap to cover finger defects. Previous studies showed that the diameter of the dorsal digital nerve at the metacarpophalangeal joint level was 0.8–1.8 mm [24], and the diameter of the proper digital nerve at the thumb interphalangeal joint level was 1.1–1.5 mm [25]. Therefore, coaptation of the dorsal digital nerve to the proper digital nerve in an end-to-end fashion could, at least in theory, provide a better axonal regeneration pathway. To the best of our knowledge, however, there is no definite answer regarding which nerve to select when performing the first dorsal metacarpal artery flap procedure. To achieve better static two-point discrimination and shorter operative time, we raised the dorsal digital nerves in the flap and made them the sensory nerves of flap without the dorsal branches of the radial and ulnar proper digital nerves of the index finger. From the results of our 137 patients, at an average of 2 years after surgery, the preservation group (*n* = 57) and non-preservation group (*n* = 80) showed no statistical differences in pain of thumb pulp, static two-point discrimination, Semmes–Weinstein monofilament score, cold intolerance in the injured finger, and patient satisfaction. Therefore, preserving the dorsal branches of the proper digital nerves of the index finger in thumb pulp reconstruction provides no obvious mid-term clinical advantage if the dorsal digital nerves were carried in the flap. Although normal sensation in the thumb pulp is not restored, the present study shows that the outcomes of our patients are superior to those previously reported in studies which did not contain dorsal digital nerves. Our technique provides improved thumb pulp sensation. We are aware that an average follow-up of 2 years may be considered relatively short. However, the outcomes of surgery would have stabilized, and recovery effected by then. We suggest that dorsal digital nerve repair should be performed, if possible, when using a first dorsal metacarpal artery flap.

This study has several strengths. First, to our knowledge this is the first study to report the clinical outcomes of the dorsal branches of the proper digital nerves of the index finger in the modified first dorsal metacarpal artery flap. Second, it carefully assessed postoperative sensory function, pain, and patient satisfaction. Third, it recruited a representative large sample of patients. Likewise, several limitations should also be noted. For example, first, the present investigation is retrospective: We are aware of the fact that such study design introduces biases which are difficult to control despite accurate surgical technique and appropriate statistical analysis. Second, results may vary if larger samples are considered, though the present is one of the largest comparative studies of its kind. Increasing the length of follow-up may influence negatively the results, though we are not aware that this has occurred in similar studies, which have shown that, by two years after the index procedure, recovery has stabilized. Third, the measuring method might not comprehensively assess all aspects of the clinical outcomes.

## Conclusions

Compared with first dorsal metacarpal artery flap carrying only dorsal digital nerves for thumb pulp reconstruction, the first dorsal metacarpal artery flap carried dorsal digital nerves and dorsal branches of the proper digital nerves of the index finger offers no mid-term advantages in clinical outcomes, such as static 2-PD, SWM, cold intolerance in the injured finger, and patient satisfaction. The modified first dorsal metacarpal artery flap innervated by the dorsal digital nerves represents a useful and reliable procedure for thumb pulp reconstruction with satisfactory flap sensation, appearance, and satisfactory function, with acceptable donor site morbidity.

## Data Availability

The datasets generated during and/or analyzed during the current study are available throughout the manuscript.

## Abbreviations

MHQ: Michigan Hand Outcomes Questionnaire
2-PD: Two-point discrimination
SWM: Semmes–Weinstein monofilament
VAS: Visual analogue scale
DDN: Dorsal digital nerves
DBPDNs: Dorsal branches of the proper digital nerves
SBRN: Superficial branch of the radial nerve
PDNs: Proper digital nerves
FDMA: First dorsal metacarpal artery
